# DeTEXT: A Database for Evaluating Text Extraction from Biomedical Literature Figures

**DOI:** 10.1371/journal.pone.0126200

**Published:** 2015-05-07

**Authors:** Xu-Cheng Yin, Chun Yang, Wei-Yi Pei, Haixia Man, Jun Zhang, Erik Learned-Miller, Hong Yu

**Affiliations:** 1 Department of Computer Science and Technology, School of Computer and Communication Engineering, University of Science and Technology Beijing, Beijing, China; 2 School of Foreign Studies, University of Science and Technology Beijing, Beijing, China; 3 School of Computer Science, University of Massachusetts Amherst, MA, USA; 4 Department of Quantitative Health Sciences, University of Massachusetts Medical School, MA, USA; Macquarie University, AUSTRALIA

## Abstract

Hundreds of millions of figures are available in biomedical literature, representing important biomedical experimental evidence. Since text is a rich source of information in figures, automatically extracting such text may assist in the task of mining figure information. A high-quality ground truth standard can greatly facilitate the development of an automated system. This article describes **D**e**TEXT**: A database for evaluating text extraction from biomedical literature figures. It is the first publicly available, human-annotated, high quality, and large-scale figure-text dataset with 288 full-text articles, 500 biomedical figures, and 9308 text regions. This article describes how figures were selected from open-access full-text biomedical articles and how annotation guidelines and annotation tools were developed. We also discuss the inter-annotator agreement and the reliability of the annotations. We summarize the statistics of the **D**e**TEXT** data and make available evaluation protocols for **D**e**TEXT**. Finally we lay out challenges we observed in the automated detection and recognition of figure text and discuss research directions in this area. **D**e**TEXT** is publicly available for downloading at http://prir.ustb.edu.cn/DeTEXT/.

## Introduction

**Fig 1:**
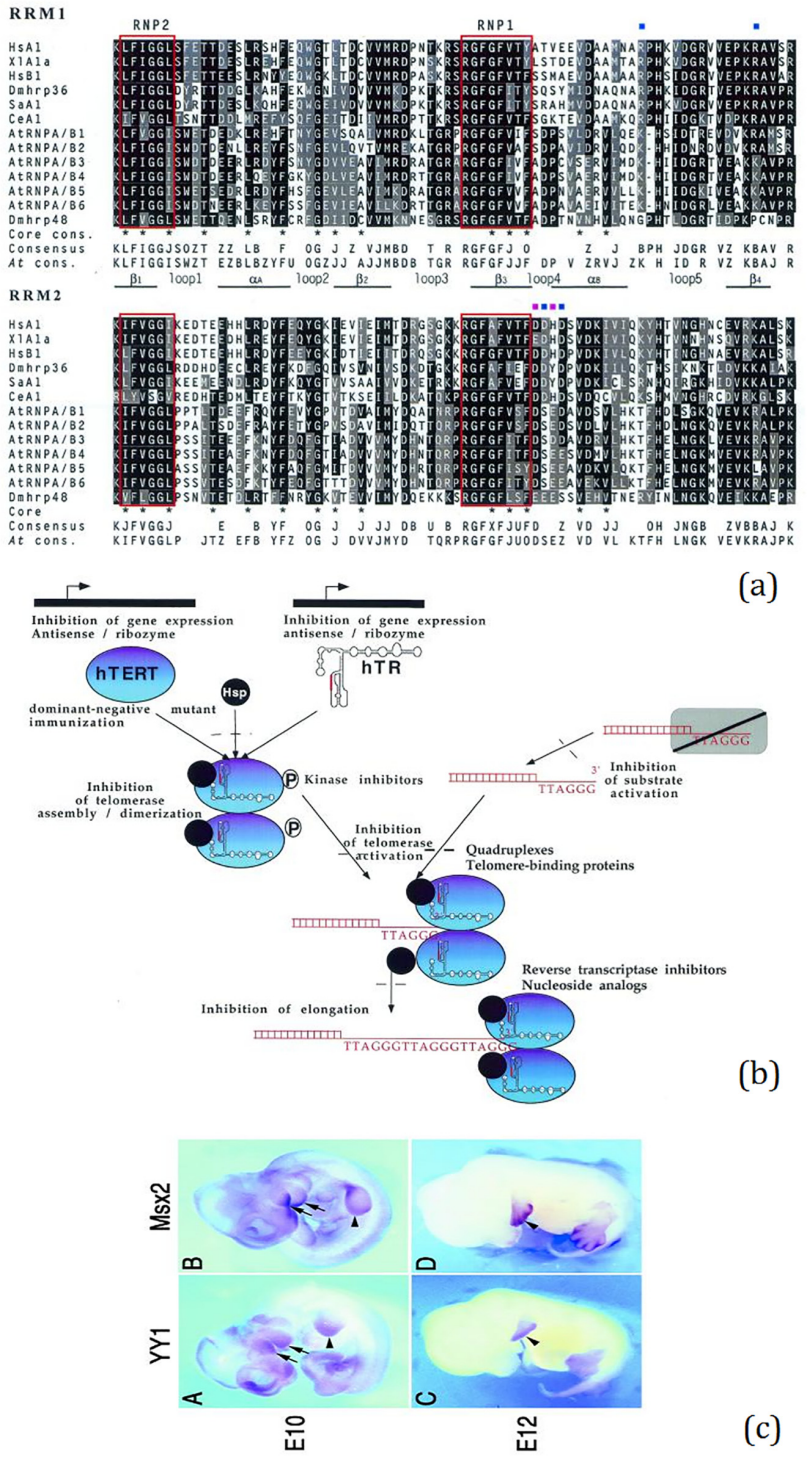
Representative biomedical figures and their texts. (a) experimental results (gene sequence), (b) research models, and (c) biomedical objects.

Figures are ubiquitous in biomedical literature, and they represent important biomedical knowledge. Fig 1 shows some representative biomedical figures and their embedded text. The sheer volume of biomedical publications has made it necessary to develop computational approaches for accessing figures. Consequently, during the last few years, figure classification, retrieval and mining have garnered significant attention in the biomedical research communities [1–12]. Since text frequently appears in figures, automatically extracting such figure text may assist the task of mining information from figures. Little research, however, has specifically explored automated text extraction from biomedical figures.

The structured literature image finder (SLIF) system applies an existing optical character recognition (OCR) system to recognize figure text and identify potential image pointers. SLIF then parses text and figures in biomedical literature by matching image pointers in images and captions [7]. Other researchers have also applied existing OCR tools to extract figure text and then incorporate the figure text for applications, e.g., image and document retrieval [5, 11]. Kim and Yu developed algorithms to improve the performance of an existing off-the-shelf OCR tool for specifically recognizing biomedical figure text [9].

Benchmark datasets have proved an invaluable resource in developing automated systems for text detection and reading. Many publicly available image datasets have had major impacts in text detection and recognition from scene images, e.g., MSRA-I [13], KIST [14], SVT [15], NEOCR [16], OSTD [17], IIIT5K Word [18], MSRA-II [19], and USTB-SV1K [20]. Using the annotated datasets as the ground truth, the International Conference on Document Analysis and Recognition (ICDAR) has held several international technical competitions on text extraction from scene images and born-digital figures by releasing a series of public benchmark datasets, i.e., ICDAR Robust Reading Competitions 2003 [21], 2005 [22], 2011 [23, 24], and 2013 [25]. Similarly, efforts to build benchmark datasets and create common ground for evaluation, including the GENIA corpus [26], the TREC Genomics [27], the BioCreative challenges [28], and the i2b2 challenges [29], have been significant in biomedical natural language processing research.

Many technologies and systems for text detection and recognition have been widely investigated and developed in the open domain for common complex images, e.g., scene images and born-digital pictures [30]. Specifically, text detection and recognition in natural scene images is a recent hot topic in the fields of Document Analysis and Recognition, Computer Vision, and Machine Learning. First, various scene text detection methods, including sliding window based methods [26, 31], connected component based methods [17, 32, 33] and hybrid methods [34], have been proposed and applied in the literature. Recently, Maximally Stable Extremal Regions (MSERs) or Extremal Regions (ERs) based methods have been the focus of many methods [35–38]. Moreover, Yin’s [38] and Kim’s [37] MSER based methods won first place in both the “Text Localization in Real Scenes” competition at ICDAR 2013 [25] and the ICDAR 2011 [24] Robust Reading Competition.

There are also significant research efforts on scene word recognition, e.g., recognition frameworks by exploiting bottom-up and top-down cues [18], recognition methods with language models [39, 40], and recognition approaches with probabilistic graphical models [41]. Specifically, “PhotoOCR”, which won first place in “Word Recognition in Real Scenes” at ICDAR 2013 [25], is built on character classification with deep neural networks and language modeling with massive training data [42]. Finally, there are also some works on end-to-end scene text recognition, e.g., word spotting based systems [43], efficient character detection and recognition based systems [35, 44], and hybrid recognition systems [45].

**Fig 2:**
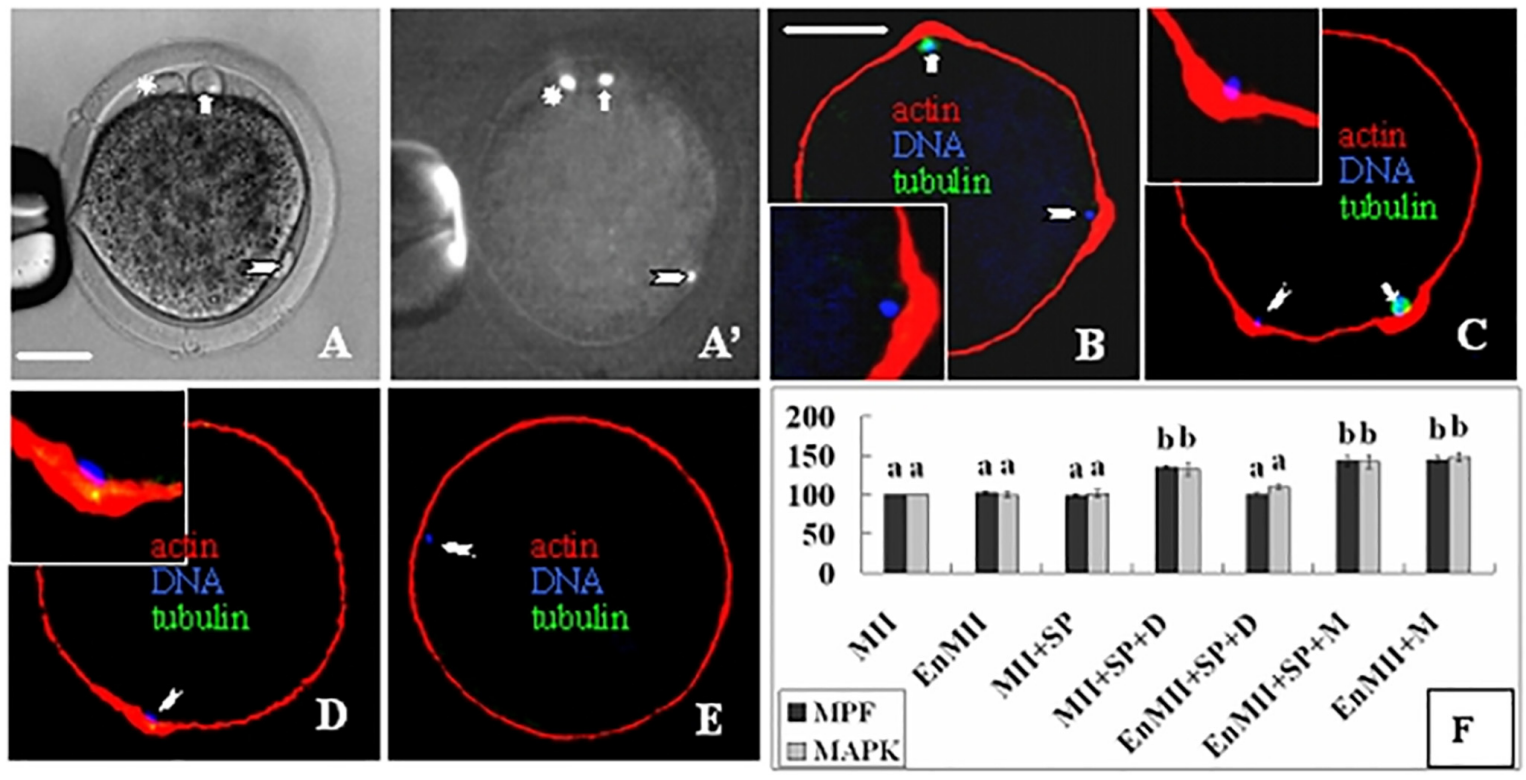
An example biomedical figure with a complex layout, color text, and irregular text arrangement.

Unlike images in the open domain, biomedical figures are highly complex and therefore present unique challenges [9]. For example, as shown in Figs 1 and 2, biomedical figures typically have complex layout, small font size, short text, specific text (e.g. gene sequence), and complex symbols. In most cases, complexity is high. As shown in Fig 2, figure text has not only come with a complex layout but also color text and irregular text arrangement. Consequently, conventional OCR technologies and systems which are typically trained on simpler open domain document images can’t deal with these challenges uniquely presented in biomedical figures. Moreover, without a high quality benchmark dataset, it would be difficult to develop and to compare different techniques for extracting figure text.

In FigTExT [9], Kim and Yu constructed a gold standard (dataset) for developing and testing figure text detection and recognition. This dataset comprises of 382 biomedical figures from 70 full-text articles randomly selected from PubMed Central. However, the dataset has significant limitations. First it is not publicly available. Secondly, authors annotated only ground truth text in figures without corresponding locations or other related information in the image. Therefore, it is not possible to use it as the benchmark to evaluate the performance of text detection and recognition technologies as done in the Document Analysis and Recognition (DAR) literature, e.g., a series of ICDAR Robust Reading Competitions.

As a result, following the general strategies in DAR, in this paper we report the development of **DeTEXT**: A database for evaluating text extraction from biomedical literature figures. Due to the complexity of biomedical figures, **DeTEXT** can be used as a common ground to evaluate text detection and recognition algorithms for complex images.

The contributions of this work are as follows. **DeTEXT** is the first figure-text annotation of biomedical literature. Giving the importance of biomedical literature and the experiments (figures), the potential impact of **DeTEXT** is huge. **DeTEXT** is large and representative. It comprises of close to ten thousands annotated text regions from hundreds of full-text biomedical articles. The annotation is rich and comprehensive. Our annotation guideline extended the existing guideline used in the open domain (e.g., the ICDAR Robust Reading Competition [25]). In our annotation, figures were annotated with not only the text region’s orientation, location and ground truth text, but also the image quality. Finally, **DeTEXT** (http://prir.ustb.edu.cn/DeTEXT/) is open-access and we will make available the fully annotated data to the public.

Moreover, compared to the datasets in the literature, **DeTEXT** has a various types of new text region features, where typical representations include blurred text, small-size characters, color text, and complex background and layouts. There are also some specific challenges from the text complexity of biomedical figures, where a large amount of short words, domain terms, upper cases, text with irregular arrangement, etc. are embedded in figures.

In summary, **DeTEXT** is the first public image dataset for biomedical literature figure detection, recognition, and retrieval that can be used as a benchmark dataset for fair comparison and technique improvement. Large scale image-text annotation including the TREC (trec.nist.gov) and CLEF (www.clef-initiative.eu) efforts have shown significant impact on the research community. In addition to being the first benchmark dataset, we will also make freely available our **DeTEXT** annotation tool, another contribution to the research community.

## Methods

In the following, we first describe how we selected figures. Then we introduce the annotation guideline and the annotation tool and describe our annotation process. Finally, several strategies for dataset separation and evaluation protocols are presented.

### A Collection of Representative Open-Access Biomedical Figures

In order to make impact in research, **DeTEXT** must be publicly available and free of licensing issues. We therefore selected open-access full-text articles and their figures from the PubMed Central (http://www.ncbi.nlm.nih.gov/pubmed). In order for **DeTEXT** to be representative, we maximized the number of figures to be annotated as well as the number of full-text articles from which the figures are included in **DeTEXT**. For this, we first randomly selected 100 articles from which we randomly selected one figure from each article. We then randomly selected an article from which we added all its figures to **DeTEXT**. We repeated this process until we reached 500, the total number of figures in **DeTEXT**. Therefore an additional 188 articles are included.

### Annotation Guideline

We have initially followed the existing guideline for image text annotation (for detection and recognition) in the open domain (e.g., ICDAR Robust Reading Competition [25]). However, we found the guideline is limited; it only requires for annotating image text with location and true text information. Figures published in the biomedical domain are complex. Studies have shown that many of them are in poor quality [9]. Moreover, some text (e.g., the mention of gene or protein names) is more semantically rich than others (e.g., panel markers) [9], we annotate not only the text region’s location, orientation, and ground truth text, but also image quality.

Following the annotation guideline [25], we annotate text region’s location and orientation information with four vertices, i.e., the left-top (LT), top-right (TR), right-bottom (RB), and bottom-left (BL) points of the text region. Some text regions can have multiple orientations (one example is illustrated in Fig 2). We also annotate orientation attributes for every text region. The “horizontal/oriented” indicates whether the text region is aligned in the horizontal (0) or oriented (or vertical, 1) direction.

We found many text regions are fragmented. An example is illustrated in Fig 1(c) with single characters “A”, “B”, “C” and “D” that usually illustrate image sections and do not carry out semantic meanings of figure content. We therefore define two additional requirements for text region inclusion. Firstly, we annotate text region that incorporates at least one or more words. Here, the “word” unit should be a character set composed of several aligned and close characters. Most text regions are in a horizontal direction; a few text regions are with multi-directions (including the vertical direction). The second requirement is word length. The length of a word to be annotated should be equal to or more than 2.

We also made changes for annotating ground truth text for a text region. In biomedical literature figures, figure texts are typically complex, including incorporating uncommon symbols. For example, a chemical formula comprises of digits, uppercase letters, superscript or subscript characters and specific symbols. Accurately identifying the location of superscript and subscript characters poses a significant challenge for human annotators. For consistent annotations, we only annotate the ground truth text of superscript or subscript characters and leave out their location information, as illustrated in Fig 2. Another rational factor for skipping the location of super- and sub-script characters is that most web-based full-text articles and documents in Database or Information Retrieval systems only provide text and characters without superscript or subscript locations. We annotate the location of other types of characters in figure text.

We assess image quality information (e.g., with blurring and noising) from the prospective of judging how difficult it would be for a human to detect and recognize the text in the annotated region. For every text region, we assign one of the following types for image quality assessment: “normal”, “blurry”, “small”, “color”, “short”, “complex_background”, “complex_symbol”, or “specific_text” (see more descriptions of “difficulty” for challenges in Section “Discussion”).

### Annotation Tool

**Fig 3:**
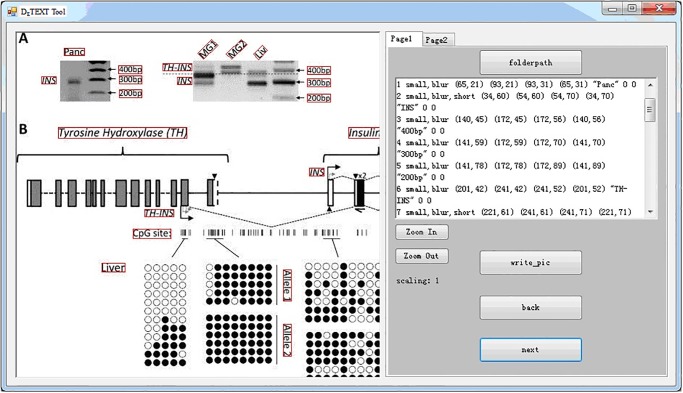
The annotation tool for DeTEXT. The figure and its annotated text regions are shown to the left. The annotated information (e.g., text and locations) is shown to the right. Functions for displaying the figure (zoom in and out), etc, are also shown to the right.

We developed an annotation tool for annotating **DeTEXT** and made it freely available from http://prir.ustb.edu.cn/DeTEXT/. We used Microsoft VS2012 (C#) to implement our tool in the Windows 32-Bit Platform. Fig 3 shows the front-end interface of the annotation tool. The figure and its annotated text regions are shown to the left. The annotated information (e.g., text and locations) is shown to the right, where “folderpath” is to open a directory of figures to be annotated, “back” and “next” are to browse previous and next figures. Functions for displaying the figure (zoom in and out) are also shown to the right. “Page1” on the right shows the annotation information for the entire figure, and “Page 2” displays detailed annotation information for each text region, including the region’s location and orientation, ground truth text and difficulty (for the image quality). In “Page 1”, “write_pic” means to start the annotation procedure. When annotating a text region, press the mouse right key on the left top corner of the region and drop to the right bottom corner. Then, “Page 2” pops up, and corresponding text region information can be easily annotated.

**Fig 4:**
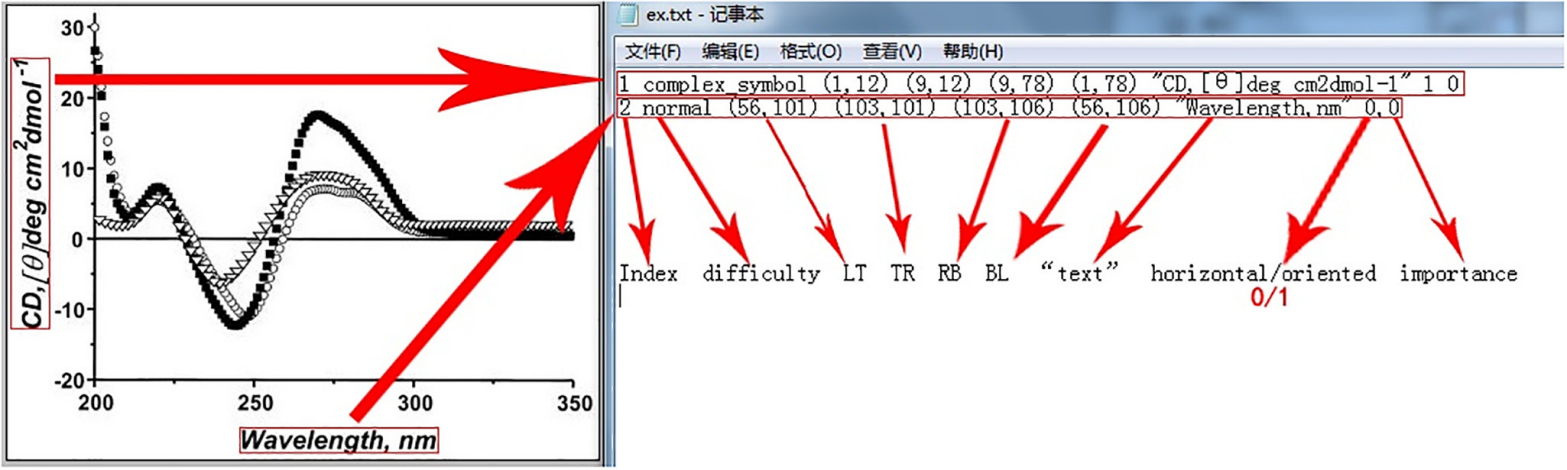
An example for the annotation information. Each figure in the database corresponds to a ground truth file (we use a “.txt” file to store the annotation information), in which each line records the information of the text in the corresponding text region.

With our annotation tool, each figure in the database corresponds to a ground truth file (we use a “.txt” file to store the annotation information), in which each line records the information of the text in the corresponding region. The format of the ground truth file (e.g., “ex.txt”) is illustrated in Fig 4.

### Annotation Process

Six annotators, all of whom are computer science graduate students in pattern recognition and image processing, completed the annotation of **DeTEXT**. We performed the annotation process with two consecutive iterations. 500 figures of the entire database are randomly divided into 5 100-figure subsets. On the first iteration, five students each independently annotated one subset. On the second iteration, each student checked one subset of figures annotated by one other student and resolve the conflicts if occurred. Our initial annotation has been an iterative process during which we refined the annotation guideline and updated the annotated data accordingly. We therefore did not report the annotation agreement. Instead, in order to measure the agreement of the inter-annotator, we asked a different annotator who followed the updated annotation guideline. This new annotator independently annotated 10 figures randomly selected from the entire database (500 figures) and we measured inter-annotator agreement with those 10 figures.

### Inter-Annotator Agreement Metrics

We simply calculated the overlap of ground truth for inter-annotator agreement of text annotation. For inter-annotator agreement with text location, we followed a metric commonly used in DAR [21]. Specifically, we compute the matching (overlapping) score between two regions, i.e., *S*_1_ and *S*_2_,

$$\begin{array}{c} fMatch\left(S_{1},S_{2}\right)=\frac{2\times Area(S_{1}\cap S_{2})}{Area\left(S_{1}\right)+Area\left(S_{2}\right)} \end{array}$$

where *S*_1_ and *S*_2_ are the regions in the original annotation and the re-annotation respectively, and *Area* is the area size of the (rectangle) region. If these two text regions in both annotations are overlapped much as *fMatch*(*S*_1_, *S*_2_) ≥ 85% then we identify these two regions are with the same location (i.e., annotation agreement for the location).

### DeTEXT Subsets Division

In the image community, a high quality annotation such as **DeTEXT** can be used as ground truth to evaluate different technologies. In order to present a fair universal evaluation database with **DeTEXT**, we present several dataset division strategies for research. First, we provided a public database of **DeTEXT** that contains all collected figures. Second, following the conventional way in the Document Analysis and Recognition field, we also divided the entire **DeTEXT** into three separate non-overlapping subsets: training, validation, and testing. We also utilized another popular strategy, cross-validation, for using the dataset.

### Evaluation Protocols of DeTEXT

There are a variety of evaluation protocols for text detection and recognition in images, most of which are based on the overlapping ratio protocol and accuracy protocol. Here, for text detection and recognition from biomedical literature figures, we followed the evaluation strategies used in a series of ICDAR Robust Reading Competitions 2003 [21], 2005 [22], 2011 [23, 24], and 2013 [25]. Specifically, we recommended the text detection and word recognition evaluation protocols used in ICDAR 2011 Robust Reading Competition (ICDAR2011), and the end-to-end text recognition evaluation protocol used in ICDAR 2003 Robust Reading Competition, for evaluating methods and systems for our **DeTEXT** dataset.

*Text detection evaluation* (with ICDAR2011 [24] protocol, DetEval [46]): This protocol comprises the area overlap and the object level evaluation. DetEval is also a software toolbox, which is publicly available at http://liris.cnrs.fr/christian.wolf/software/deteval/index.html. First, from the two sets *D* and *G* of detected rectangles (regions) and ground truth rectangles, we can construct two recall and precision matrices *σ* and *τ* of the area overlap where the rows of the matrices correspond to the ground truth rectangles and the columns correspond to the detected rectangles [47]. Here, the values of the *i* ^*th*^ row and *j* ^*th*^ column of these two matrices are

$$\begin{array}{c} \sigma_{ij}=R_{AR}\left(G_{i},D_{j}\right)=\frac{Area(G_{i},D_{j})}{Area(G_{i})} \end{array}$$

$$\begin{array}{c} \tau_{ij}=P_{AR}\left(G_{i},G_{j}\right)=\frac{Area(G_{i},D_{j})}{Area(D_{i})} \end{array}$$

where *Area* is the area size of the rectangle region. Then, the two rectangles are decided as matched ones if

$$\begin{array}{c} \sigma_{ij}>t_{r}=0.8,\;\;\;\;\tau_{ij}>t_{p}=0.4 \end{array}$$

By supporting one-to-one, one-to-many, and many-to-one matches among ground-truth objects and detections, this evaluation strategy deals with over-split or over-merge of detections [46]. Based on this matching strategy, the recall and precision measures in one image can be defined as

$$\begin{array}{c} Recall\left(G,D,t_{r},t_{p}\right)=\frac{\sum_{i}Match_{G}\left(G_{i},D,t_{r},t_{p}\right)}{\left|G\right|} \end{array}$$

$$\begin{array}{c} Precision\left(G,D,t_{r},t_{p}\right)=\frac{\sum_{j}Match_{D}\left(D_{j},G,t_{r},t_{p}\right)}{\left|D\right|} \end{array}$$

where *Match*_*G*_ and *Match*_*D*_ are functions by considering different types of matches. These functions are defined as

$$\begin{array}{c} Match_{G}\left(G_{i},D,t_{r},t_{p}\right)=\{\begin{array}{cc} 1\;\;\;\;\;\;\;\;\;\text{if}\;G_{i}\;\text{matches}\;\text{against}\;\text{a}\;\text{single}\;\text{detected}\;\text{rectangle}, & \\ 0\;\;\;\;\;\;\;\;\;\text{if}\;G_{i}\;\text{does}\;\text{not}\;\text{match}\;\text{against}\;\text{any}\;\text{detected}\;\text{rectangle}, & \\ f_{sc}\left(k\right)\;\;\text{if}\;G_{i}\;\text{matches}\;\text{against}\;\text{several}\;\text{(}k\text{)}\;\text{detected}\;\text{rectangles}. \end{array} \end{array}$$

$$\begin{array}{c} Match_{D}\left(D_{j},G,t_{r},t_{p}\right)=\{\begin{array}{cc} 1\;\;\;\;\;\;\;\;\;\text{if}\;D_{j}\;\text{matches}\;\text{against}\;\text{a}\;\text{single}\;\text{detected}\;\text{rectangle}, & \\ 0\;\;\;\;\;\;\;\;\;\text{if}\;D_{j}\;\text{does}\;\text{not}\;\text{match}\;\text{against}\;\text{any}\;\text{detected}\;\text{rectangle}, & \\ f_{sc}\left(k\right)\;\;\text{if}\;D_{j}\;\text{matches}\;\text{against}\;\text{several}\;\text{(}k\text{)}\;\text{detected}\;\text{rectangles}. \end{array} \end{array}$$

where *f*_*sc*_(*k*) is set as a constant (0.8). In the case of *N* images with $\bar{G}=\{G^{1},...,G^{k},...,G^{N}\}$ and $\bar{D}=\{D^{1},...,D^{k},...,D^{N}\}$, text region recall and precision are defined as

$$\begin{array}{c} Recall\left(\bar{G},\bar{D},t_{r},t_{p}\right)=\frac{\sum_{k}\sum_{i}Match_{G}\left(G_{i}^{k},D^{k},t_{r},t_{p}\right)}{\sum_{k}\left|G^{k}\right|} \end{array}$$

$$\begin{array}{c} Precision\left(\bar{G},\bar{D},t_{r},t_{p}\right)=\frac{\sum_{k}\sum_{j}Match_{D}\left(D_{j}^{k},G^{k},t_{r},t_{p}\right)}{\sum_{k}\left|D^{k}\right|} \end{array}$$

Finally, f-score is easily calculated as

$$\begin{array}{c} f_{score}=\frac{1}{0.5/Precision+0.5/Recall}. \end{array}$$

Please note that for the rotated text detection region, we will first correct the rotated rectangle to the horizontal rectangle, and then use this protocol for evaluating.

*Word recognition evaluation* (with ICDAR 2011 [24] protocol): Word recognition is usually and simply evaluated by

$$\begin{array}{c} Accuracy=|C|/|G|, \end{array}$$

where *C* and *G* are the correctly recognized word set and ground truth set respectively.

*End-to-end text recognition evaluation* (with ICDAR 2003 [21] protocol): This protocol uses the standard measures of precision, recall and f-score to evaluate the performance of the end-to-end system, where it rates the quality of match between a target and the estimated rectangle, and defines a strict notion of match between the target and the estimated words: the rectangles must have a match score greater than 0.5 and the word text must match exactly. The match score between two bounding rectangles of text objects is defined as the ratio between the area of intersection and that of the minimum bounding rectangle containing both rectangles. Suppose *M*, *D* and *G* are the set of correctly recognized and location matched text regions, the set of all detected regions, and the set of ground truth regions respectively, the definitions of precision and recall are

$$\begin{array}{c} Precision=|M|/|D|,\;\;\;\;Recall=|M|/|G|, \end{array}$$

and f-score is correspondingly computed as

$$\begin{array}{c} f_{score}=\frac{1}{0.5/Precision+0.5/Recall}. \end{array}$$

Similar to the evaluation on the important figure text in [9], we can conveniently evaluate text detection, word recognition, and end-to-end text recognition on the subset of the important figure text according to the corresponding text importance in the full article. Moreover, in DeTEXT, we are also able to measure the performances of text detection, word recognition, and end-to-end text recognition methods on the subset of the figure text according to the corresponding difficulty for the image quality of the figures.

## Results

### Inter-Annotator Agreement

Table 1 shows the annotation agreement results (i.e., the same location by *fMatch*(*S*_1_, *S*_2_) ≥ 85% and the same annotated text in both annotations) of the 10 double-annotated figures (see the above subsection “Annotation Process”). Using the first run annotation as the standard, we found that the agreement of the second run annotation is over 97% in both ground truth text and location. Actually, with Table 1, the text and location agreement percentages are same, and are calculated as

$$\begin{array}{c} \frac{176}{\max\{181,189\}}=97.24\% \end{array}$$

**Table 1:** The annotation agreement of the 10 figures randomly selected.

	Original annotations	Re-annotations
Number of text regions	181	189
Number of text regions which have *the same annotated text* in both annotations	176	176
Number of text regions which have *the same location* in both annotations	176	176

**Fig 5:**
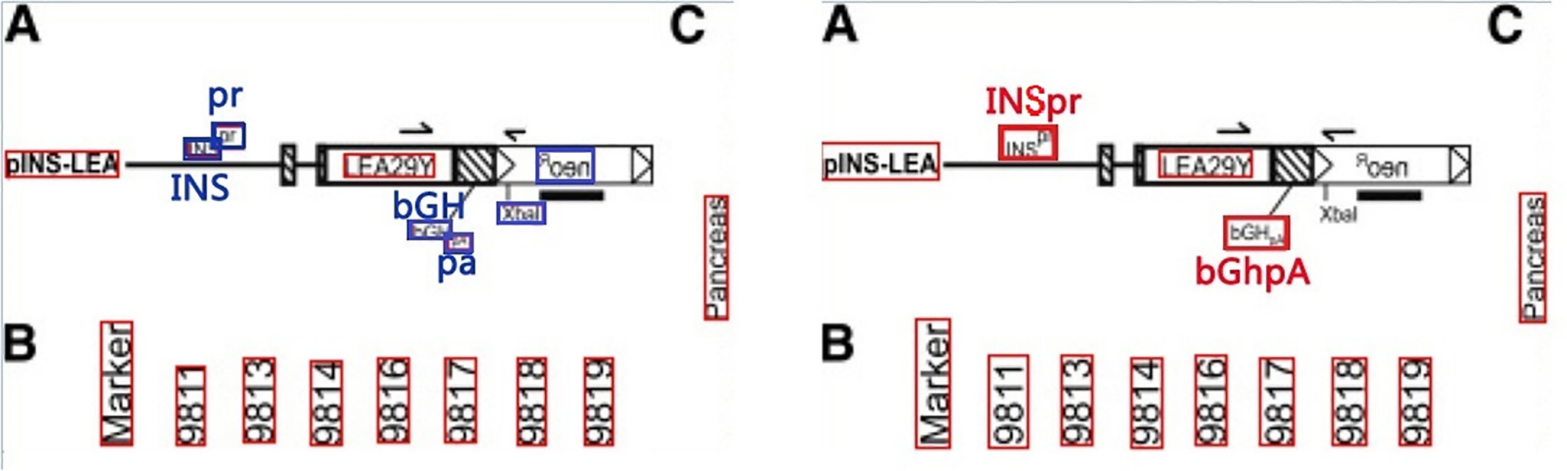
Disagreed examples between the original annotation and the re-annotation, where thick blue and red boxes are text regions with inconsistent annotations.

We manually analyzed the inconsistent annotations. A few examples are shown in Fig 5, in which thin red boxes are agreed annotations while thick blue boxes and thick red boxes are in disagreement, representing the original annotation and the re-annotation respectively. Fig 5 also shows cases where ground truth text differs.

There are two main reasons for the disagreement, which correspond to two types of text regions, i.e., text with low image quality and text with domain-specific terms. First, although the quality of images overall is reasonable, in some cases, text regions are blurry and small which may be overlooked by the annotators. In addition, domain-specific terms in biomedical literature (e.g., “INSpr” and “bGHpA” in Fig 5) are also challenging. Despite the challenges, the overall agreement is high and therefore we consider **DeTEXT** a high-quality annotated corpus for biomedical figures.

### Data Statistics

**Fig 6:**
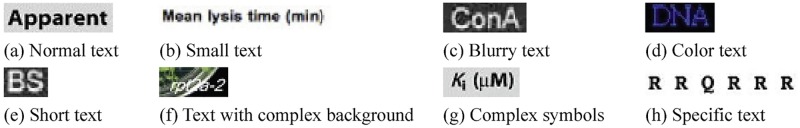
Region samples of different categories.

As described previously, **DeTEXT** comprises of a total of 500 open-access publicly available figures that appear in 288 full-text articles randomly selected from PubMed Central. **DeTEXT** is composed of a total of 9308 text regions which are finely annotated. It is a large-scale dataset for text extraction from images and figures, as in the open domain many publicly available image datasets (e.g. ICDAR Robust Reading Competition datasets) only have about 2000 text (word) regions. Table 2 shows the annotation statistics by different text regions, and Fig 6 shows region samples of different categories. As shown in Table 2, “short” is the most common type of region, accounting for 46.8% (4,354/9,308) of all annotated text regions. “Normal” follows the second, accounting for 37.8% (3,519/9,308) of all annotated text regions. “Small”, “blurry”, “color”, “complex_background”, “complex_symbol”, and “specific_text” account for the remaining text regions.

**Table 2:** Statistics of text (word) regions and figures with different categories.

Text region category	NO. of regions (%)	NO. of figures (%)
Normal	3519 (37.8%)	424 (84.8%)
Small	2419 (26.0%)	151 (30.2%)
Blurry	1118 (12.0%)	65 (13.0%)
Color	293 (3.1%)	39 (7.8%)
Short	4354 (46.8%)	379 (75.8%)
Complex_background	670 (7.2%)	86 (17.2%)
Complex_symbol	240 (2.6%)	75 (15.0%)
Specific_text	74 (0.8%)	14 (2.8%)

We further counted the number of text regions belonging to multiple categories as shown in Table 3. The most common text regions are “small”+“short”, followed by “small”+“blurry” and “blurry”+“short”.

**Table 3:** Statistics of text (word) regions and figures with combination of categories.

Combination of region categories	NO. of regions	NO. of figures
short, complex_symbol	71	18
small, short	1786	126
complex_background, complex_symbol	23	9
color, short	96	22
small, blurry	858	47
small, blurry, short	485	33
short, complex_background	279	48
blurry, short	603	44
small, complex_symbol	19	9
color, specific_text	35	2
small, blurry, complex_symbol	7	5
small, complex_background	106	13
blurry, complex_symbol	14	7
small, short, complex_background	47	8
color, complex_background	81	16
color, short, complex_background	24	9
small, color, short	10	4
color, complex_symbol	2	1
small, color	28	7
small, blurry, complex_background	43	4
small, blurry, short, complex_background	9	2
short, complex_background, complex_symbol	5	2
small, short, complex_symbol	5	2
blurry, complex_background, complex_symbol	2	1
blurry, short, complex_background	11	3
small, color, complex_background	15	2
complex_background, specific_text	3	2

We also annotated orientation attributes (“horizontal/oriented”) for every text region. As shown in Table 4, over 9% (847/9,308) of all annotated text regions have rotated text. Table 4 also shows that there are both horizontal and oriented text regions in some figures (see Fig 2 as a common case).

**Table 4:** Statistics of text (word) regions with orientation attributes.

Orientation attribute	NO. of regions	NO. of figures
Horizontal	8461	492
Oriented	847	268
*Total*	*9308*	*500*

Since biomedical figures can be classified into five different types(i.e., Gel-image, Image-of-thing, Graph, Model, and Mix) [48]. Table 5 shows the statistics of images among image types. Here, *Gel-image* consists of gel images (e.g., DNA, RNA and protein); *Image-of-thing* refers to pictures of existing objects such as cells, tissues, organs, and equipments; *Graph* consists of bar chart, column charts, line charts, plots and other drawn graphs; *Model* demonstrates a biological process, a chemical or cellular structure, or an algorithm framework; and *Mix* refers to a figure that incorporates two or more other figure types. In **DeTEXT**, there are 16, 46, 232, 124, and 82 images for *Gel-image*, *Image-of-thing*, *Graph*, *Model*, and *Mix* respectively, which will be sufficient to represent general situations for text extraction from different biomedical figures.

**Table 5:** Statistics of biomedical figures with five different types.

	*Gel-image*	*Image-of-thing*	*Graph*	*Model*	*Mix*
NO. of figures	16	46	232	124	82

### Data Subsets for Evaluation

First, the researchers can download this entire dataset of **DeTEXT** with 500 figures, and these resources may be altered, amended or annotated in any way for facilitating related research issues.

Second, we also got three separate non-overlapping subsets: training, validation, and testing. Details are shown in Table 6.

**Table 6:** Training, validation, and testing sets of DeTEXT.

Subset	NO. of figures	NO. of articles	Remarks
Training set	100	100	Select one figure for each article.
Validation set	100	45	Randomly select 45 articles and include all common figures in these articles from the remaining dataset without the training set.
Testing set	300	143	The remaining subset after selecting the validation set.
*Total*	*500*	*288*	

The training set comprises 100 figures from 100 articles (each figure from one article), maximizing the number of both figures and articles used for training. The validation set is composed of 100 figures from 45 articles randomly selected from the remaining dataset after the construction of the training set is finished. The testing set is the remaining subset after the construction of the training and validation sets are completed. It comprises 300 figures from 143 articles.

Similar to the entire dataset (in Table 3), we also presented the annotation statistics by different text regions and figures with different categories of these three separate non-overlapping subsets (training, validation, and testing sets) in Table 7. From Table 7, we can see that training, validation, and testing sets have similar distributions of regions and figures with different text region categories (challenges for text recognition).

**Table 7:** Statistics of text regions and figures with different categories on the training, validation, and testing sets.

Text region category	NO. of regions			NO. of figures		
	Training	Validation	Testing	Training	Validation	Testing
Normal	731	597	2191	76	83	265
Small	703	483	1233	37	36	78
Blurry	638	8	472	28	1	36
Color	52	11	230	7	3	29
Short	964	780	2610	81	63	235
Complex_background	270	126	294	24	15	47
Complex_symbol	112	20	128	33	5	42
Specific_text	10	8	56	2	5	7

Third, for the cross-validation separation strategy, if we take all of the images (actually the entire **DeTEXT** database), and do 5-fold cross validation, then for each fold we can use 400 for training and 100 for testing. As a result, we constructed 5-fold and 10-fold cross validation datasets which are public and available at http://prir.ustb.edu.cn/DeTEXT/.

Finally, according to the categories of biomedical images (i.e., Gel-image, Image-of-thing, Graph, Model, and Mix), **DeTEXT** is grouped into these 5 image categories, i.e., 5 subsets. Hence, only one type of images can be chosen for the evaluation.

## Discussion

Throughout the **DeTEXT** annotation, we found unique challenges for automatically detecting text from figures. As shown in Tables 2, 3 and 4, only 37.8% text regions are normal. In most cases, text is small (26.0%), blurry (12.0%), short (46.8%), embedded in complex background (7.2%), with different orientations (9.1%), and with a combination of multiple aforementioned challenges. For example, as shown in Table 3, 19.2% (1,786/9,308) figure text is both small and short, and 9.2% (858/9308) figure text is both small and blurry. All these issues are significant challenges to figure text recognition, and most conventional OCR technologies would likely fail. In the following we focused on the discussion of challenges from image quality and complex images in both the open domain and the specific domain (biomedical figures), and challenges from text regions themselves in the specific domain. Finally, we also discussed some issues of the size of **DeTEXT**, and presented some possible future research directions.

### Image Quality, Complex Images and Complex Background

We believe that figure image quality poses significant challenges for automatic text detection and recognition. In addition, complex images have many common challenges due to environment complexities, flexible acquisitions, and text variations [30]: background complexity, blurring and degradation, aspect ratios of text, various text fonts, and image distortion.

Biomedical literature figures are sometimes displayed with a low resolution. In a low-resolution image, text is always composed of blurry and small-size characters. In our annotation (training, validation, and testing) datasets, there are about a quarter of figures with blurry text or / and small-size characters (see examples in Fig 1).

Layout complexity is one of the characteristics of biomedical figures. As shown in Figs 1 and 2, figures compose of different objects, including experimental results, research models, and biomedical objects with different targets, patterns and presentations. Consequently, they form a complex layout for figure representation. For example, Fig 2 is simultaneously composed of biomedical objects, experimental results, different graphs, rotated and color text. This complex layout is a big challenge not only for image processing but also for text extraction.

In summary, challenges from image quality and complex images in both the open domain and the specific domain mainly include blurred text, small-size character, color text, and complex background and layout, which are described in details in the following.

Blurred text (“blurry”): Because of the limitation of the file size, or the incorrect handling of the figure itself, it is common to see blurred figures. It degrades the quality of text images. The common influence of blurring and degradation is that they always reduce characters’ sharpness and introduce touching characters (see Fig 1(B)), which makes text detection, character segmentation, and word recognition very difficult.

Small-size character (“small”): Generally, literature figures have limited space for text insertion and presentation. Consequently, authors often use a small font size when embedding text. Small font size, however, often lowers both image quality and contrast, as in Fig 1(B), serving as one main error source. Moreover, sometimes there are also some oversized characters in figures. Characters of various fonts and sizes have large within-class variations, and could form many pattern subspaces, making it difficult to perform good segmentation and recognition.

Color image / text (“color”): In order to clearly and discriminatively present information and objects, there is plenty of color text or/and color background in figures (see Fig 2). Color variation introduces challenges in text localization, segmentation and recognition.

Complex background and layout (“complex_background”): In biomedical literature figures, there are lots of experimental results, research models and biomedical objects with different representations and frequently intertwined text and image content(examples are given in Figs 1(a), 1(B) and 2). These objects and their embedded text contribute to the layout complexity and make it difficult to localize and segment text.

### Text Complexity

In the specific domain of biomedical figures, there are a large amount of short words, domain terms, upper cases, text with irregular arrangement, etc. This text complexity also bring several significant challenges for figure text recognition. For example, irregular text arrangement is a common characteristic in biomedical figures (see Figs 1 and 2). The figure is the precise, concise description of one idea (or content) in a paper. In a limited-scale figure, text is always arranged with a wide range of sizes, orientations, and locations.

In summary, challenges from texts themselves in the specific domain mainly include short words, complex symbols, specific text, and oriented text, which are described in details in the following.

Short word (“short”): There are plenty of short words (two or three characters) in figures (see Figs 1(c) and 2). Two or three characters are always difficult for text grouping and text classification in the text detection stage. Moreover, some noise regions have similar structures and appearances with short words.

Complex symbol (“complex_symbol”): In biomedical literature figures, there is plenty of complex text with complex and specific symbols, e.g., chemical formula, molecular, and abbreviations (see Fig 2). A chemical formula is always composed of digits, uppercase letters, superscript or subscript characters, and specific symbols. Besides the big challenge for character and word recognition, it is also very difficult for layout analysis and text detection.

Specific text (“specific_text”): There are several specific texts in biomedical figures. The two most common ones are gene sequence and linked terms [9]. One gene sequence is composed of several characters, which are always shown in tables (see Fig 1(a)). However, the spacing between characters is sometimes small and sometimes large. Consequently, it is very difficult to detect and locate the text region of the whole sequence. But a whole gene sequence unit is very important, as well as enjoying a high priority, for figure retrieval and text mining.

Another issue is rotated (oriented) text. Multi-orientation text is always embedded in literature figures in order to compact representation and beautiful arrangement. Two common cases are the vertical text along the Y-axis (Fig 3), and the oriented text (with a long text) along the X-axis in plot and histogram figures (Fig 5). However, most existing methods have focused on detecting horizontal or near-horizontal texts in images and figures due to the challenging issues for detecting multi-orientation text. The fundamental difficulty is that the text line alignment feature can no longer be used to regularize the text construction process. However, most current clustering- or rule-based methods always rely on such information for character grouping and line construction [17, 34, 38, 44] because the bottom alignment is the key and most stable feature for text lines [38]. Another challenge is that in arbitrary orientations, it is complicated to determine numerous empirical rules and to train robust character and text classifiers for text detection and recognition.

Table 8 summarizes all aforementioned common and notable challenges (“difficulties”) for text detection and recognition from biomedical literature figures.

**Table 8:** Challenges for text detection and recognition from biomedical literature figures.

Challenges	Sub Categorization	Difficulty
From image quality and complex images	Blurred text	“blurry” (see Fig 1(b))
	Small-size character	“small” (see Fig 1(b))
	Color image / text	“color” (see Fig 2)
	Complex background and layout	“complex_background” (see Fig 2)
From text complexity	Short word	“short” (see Fig 1(c))
	Complex symbol	“complex_symbol” (see Fig 4)
	Specific text	“specific_text” (see Fig 1(a))
	Oriented text	“oriented” (see Fig 2)

### Database Size and Annotation Effort

As described previously, **DeTEXT** comprises of a total of 9308 text regions from 500 figures of 288 full-text articles. Significant amount of annotation work has been put forth in the biomedical domain. For example, two highly successful text-based evaluation efforts, the BioCreAtIvE (http://biocreative.sourceforge.net/index.html) and the i2b2 (https://www.i2b2.org/) both have the annotated corpora at the scale of a hundred or a few hundred. A five-year annotation effort supported by NIH resulted in 97 annotation of full-text articles [49]. We have also demonstrated that careful annotations of hundreds or less articles can lead to meaningful biomedical knowledge discoveries [10]. Since biomedical images can be classified mainly into five types [48], with thousands of text regions annotated for each image type, we are confident that our annotation data size is sufficient as a benchmark dataset.

### Future Work

As we know, hundreds of millions of figures are available in biomedical literature, representing important biomedical experimental evidence. Since text richly appears in figures, text extraction (detection and recognition) from figures is an important step for applications of figure text and figure mining in biomedical literature. Consequently, one future work is to develop automated systems to detect and recognize text in biomedical figures. Unlike images in the open domain, biomedical figures are highly complex and therefore present unique challenges. DeTEXT provides a high quality benchmark dataset for exploring automated text extraction from biomedical figures in both biomedical informatics and document analysis and recognition fields. Another possible work is to perform biomedical figure search which combines a variety of information from both figure captions, full-text article and also the text embedded in its figure. Again, DeTEXT along with its full articles provide a good resource for investigating such topics in both biomedical informatics and information retrieval fields.

### Conclusion

In this paper, we released the first public image dataset for biomedical literature figure text detection and recognition, **DeTEXT**: a Database for **E**valuating **TEXT**-extraction from biomedical literature figures. Similar to the figure dataset in FigTExT [9] but with a larger number of figures and articles, **DeTEXT** is composed of 500 typical biomedical literature figures existing in about 300 full-text articles randomly selected from PubMed Central. Moreover, similar to the image dataset in the recent ICDAR Robust Reading Competition [25] but with much richer information, images in **DeTEXT** are annotated with not only the text region’s orientation, location and ground truth text, but also the image quality that is essential for technology study, error analysis and application investigation. Meanwhile, we also recommended the text detection and word recognition evaluation protocols for our **DeTEXT** dataset. The next tasks are how to detect and recognize figure text in this dataset, and how to retrieve biomedical literature figures with figure text extraction. We hope our continuous efforts will help to improve figure classification, retrieval and mining in the literature.
